# Correction: Heterogeneity in surface sensing suggests a division of labor in *Pseudomonas aeruginosa* populations

**DOI:** 10.7554/eLife.59154

**Published:** 2020-05-26

**Authors:** Catherine R Armbruster, Calvin K Lee, Jessica Parker-Gilham, Jaime de Anda, Aiguo Xia, Kun Zhao, Keiji Murakami, Boo Shan Tseng, Lucas R Hoffman, Fan Jin, Caroline S Harwood, Gerard CL Wong, Matthew R Parsek

Armbruster CR, Lee CK, Parker-Gilham J, de Anda J, Xia A, Zhao K, Murakami K, Tseng BS, Hoffman LR, Jin F, Harwood CS, Wong GCL, Parsek MR. 2019. Heterogeneity in surface sensing suggests a division of labor in Pseudomonas aeruginosa populations. *eLife* **8**:e45084. doi: 10.7554/eLife.45084.

Published 10, June 2019

This change of correction is to add Fan Jin and Gerard Wong as co-corresponding authors. This will not affect the order the authors appear in the reference.

This change is justified in that Dr Wong (Figure 4) and Dr Jin (Figure 5) both applied crucial data that warrants their co-authorship. The manuscript couldn’t have been published without this data and represents a significant amount of work.

The co-authors on the manuscript have all agreed to this change.

The article has been corrected accordingly.

